# Mutations introduced in susceptibility genes through CRISPR/Cas9 genome editing confer increased late blight resistance in potatoes

**DOI:** 10.1038/s41598-021-83972-w

**Published:** 2021-02-24

**Authors:** Nam Phuong Kieu, Marit Lenman, Eu Sheng Wang, Bent Larsen Petersen, Erik Andreasson

**Affiliations:** 1 Department of Plant Protection Biology, Swedish University of Agricultural Sciences, Alnarp, Sweden; 2 Department of Plant and Environmental Sciences, University of Copenhagen, Thorvaldsen’s vej 40, 1871 Frederiksberg C, Denmark

**Keywords:** Molecular biology, Plant sciences

## Abstract

The use of pathogen-resistant cultivars is expected to increase yield and decrease fungicide use in agriculture. However, in potato breeding, increased resistance obtained via resistance genes (R-genes) is hampered because R-gene(s) are often specific for a pathogen race and can be quickly overcome by the evolution of the pathogen. In parallel, susceptibility genes (S-genes) are important for pathogenesis, and loss of S-gene function confers increased resistance in several plants, such as rice, wheat, citrus and tomatoes. In this article, we present the mutation and screening of seven putative S-genes in potatoes, including two DMR6 potato homologues. Using a CRISPR/Cas9 system, which conferred co-expression of two guide RNAs, tetra-allelic deletion mutants were generated and resistance against late blight was assayed in the plants. Functional knockouts of *StDND1*, *StCHL1,* and DMG400000582 (*StDMR6-1*) generated potatoes with increased resistance against late blight. Plants mutated in *StDND1* showed pleiotropic effects, whereas *StDMR6-1* and *StCHL1* mutated plants did not exhibit any growth phenotype, making them good candidates for further agricultural studies. Additionally, we showed that DMG401026923 (here denoted *StDMR6-2*) knockout mutants did not demonstrate any increased late blight resistance, but exhibited a growth phenotype, indicating that *StDMR6-1* and *StDMR6-2* have different functions. To the best of our knowledge, this is the first report on the mutation and screening of putative S-genes in potatoes, including two DMR6 potato homologues.

## Introduction

Potatoes (*Solanum tuberosum* L.) are the third-fourth most important staple crop worldwide with 450 million tons produced in 2018 (www.fao.org) and are a major and irreplaceable part of the human diet in some countries^1^. Potatoes have potential for extraordinarily high yield, have a high nutritional value, and are a good source of energy, minerals, protein, fats, and vitamins^2^. However, potato crops are affected by pests and many diseases, such as late blight, early blight, bacterial wilt, potato blacklegs, Colorado potato beetles, and cyst nematodes (https://cipotato.org/crops/potato/potato-pests-diseases/).

Late blight is the most serious disease of potato crops worldwide. It is caused by the oomycete pathogen *Phytophthora infestans*, which can infect the leaves, stems, and tubers of potato plants. Under favourable conditions like moderate temperatures and moderate to high humidity, an unprotected potato field with a late blight susceptible cultivar can be destroyed in matter of days by *P. infestans* infection^3^. The control of late blight disease is mainly dependent on the use of fungicides and to a less degree resistant potato varieties. Normally, several fungicide sprays are applied during a cropping season to control late blight disease^4^. Resistant potato crop varieties require less fungicide use; therefore, use of resistant crops is a more sustainable method for control of late blight. Late blight-resistant potato varieties have been developed for more than a century by introgression of resistance genes (R-genes) from wild *Solanum* species^5^. However, virulent races of *P. infestans* have rapidly evolved to overcome all 11 major R-genes introduced from *S. demissum*^3^. Recently, breeders have tried to combine several R-genes from different wild *Solanum* relatives to increase late blight resistance in potatoes^6,7^. However, classical breeding by recurrent selection is time-consuming as well as complicated in tetraploid potatoes.

Another type of resistance, based on the loss-of-function of a susceptibility gene (S-gene), has more recently been described. S-genes are utilized by the pathogen during colonization and infection. Therefore, the knockout of S-genes may induce recessive resistance in plants^8^. One typical S-gene is *MLO* (Mildew Locus O), which was originally characterized in spring barley in the 1940s and later used in European plant breeding programs in the 1970s. Because it provides nonspecific durable resistance in the field, MLOs have been used in a wide range of plant crops such as apples, barley, cucumbers, grapevines, melons, peas, tomatoes, and wheat^9–11^. Based on biological function, S-genes have been divided into three groups^12,13^. The first group includes genes needed for host recognition by the pathogen. One example is GLOSSY 11 in maize^12^. The second group comprises genes that support pathogen demands, such as SWEET sugar transporters. The third group includes genes that control plant defence responses. Many S-genes encode negative regulators of plant defence responses, such as *DMR6*, *TTM2,* and *LSD1*. Using RNAi silencing, Sun et al. (2016) identified some S-genes in potatoes, including *StDND1* and *StDMR6* that upon knockdown showed enhanced late blight resistance. However, downregulation of homologous genes can cause undesirable phenotypes, or silencing of the introduced transgene may produce uneven results using the RNAi method. Finally, RNAi approaches are clearly classified as genetically modified organisms (GMOs).

Recently, genome editing technologies have progressed and become powerful genetic tools for increasing pathogen resistance in plants^14^. These technologies include the use of transcription activator-like effector nucleases (TALENs) or clustered regularly interspaced short palindrome repeats (CRISPR)/CRISPR-associated protein 9 (Cas9)^14,15^. CRISPR-Cas is the preferred genome editing tool because of both the versatile and easy design, which only requires replacement of the sgRNA to confer new target specificity. This makes it cost and labour effective, as well as giving it the ability to produce transgene-free offspring^14,16^. Recently, CRISPR-Cas has been used to knock out *elF4E* in cucumbers, *SWEET14* in rice, *CsLOB1* in citrus, and *DMR6-1* or *JAZ2* in tomatoes^17^, but it has not been applied in tetraploid potatoes for enhanced disease resistance^18^. In potato, gene editing has been used for improving of tuber quality traits^16,19,20^.

Most potato cultivars used commercially are tetraploid and rarely produce berries^21^. Therefore, increased resistance of these cultivars by traditional breeding methods is laborious, and finding natural or chemical mutants, which are mutated in all four alleles, is exceedingly difficult and cumbersome. Čermák et al. (2017) developed a whole array of CRISPR-Cas9 vectors, which were used to produce deletion mutants on diploid plants, such as tomatoes and *Medicago*. Additionally, larger CRISPR/Cas mediated deletions may easily be scored by PCR with primers specific to or flaking the target region^22,23^.

To produce late blight resistance potato cultivars in the future, we initiated the first step of screening putative S-genes in potatoes. Based on predicted gene function, target candidates in potatoes were selected using the following criteria: pathogen resistance phenotype, small gene family size, and different gene functions and pathways. Seven putative S-genes from the literature were selected (Table 1), and plants with mutated genes were generated by CRISPR/Cas9 and analysed for late blight resistance. Our results demonstrated that *StDMR6-1* and *StCHL1* are promising S-gene candidates for generating increased late blight resistance in potatoes.

**Table 1:** Selected putative S-genes in potatoes.

Candidate Gene name	Pathogen/host	Function	Non-pathogen related phenotypes	Reference	Potato gene
*MLO (several species)*	*Phytophthora palmivora* /barley *H. arabidopsidis* / Arabidopsis—powdery mildew/barley, wheat, cucumber, tomato	Encodes a seven transmembrane protein involved in vesicle transport and callose deposition	Premature senescence (Barley, wheat, Arabidopsis) Reduced plant size (pepper) None (tomato, pea, tobacco, melon, apple)	^9,10,35–37^	*StMLO1* (CP055237.1:44,321,938–44,323,981 in Solyntus)
*AtHDS*	*P. syringae* / *A. thaliana*	Encodes 1-hydroxy-2-methyl-2-butenyl 4-diphosphate synthase involved in salicylic acid hormone signalling	Albino phenotype and seedling lethality when homozygous for the deletion	^38^	DMG400008050
*AtTTM2*	Hyaloperonospora sp./ *A. thaliana*	Encodes a triphosphate tunnel metalloenzyme; a negative regulator of defence responses	None	^39^	DMG400025117
*StDND1*	*P. infestans*/potato *H. parasitica* / *A. thaliana*	Encodes a cyclic nucleotide-gated ion channel protein which has a role in conducting Ca^2+^ into plant cells	Necrotic spots on older leaves	^12,40,41^	DMG400001441
*StCHL1* (bHLH7)	*P. infestans* / Tobacco, tomato	Encodes a transcription factor, involved in brassinosteroid (BR) hormone signalling, which interacts with the RXLR effector AVR2	Unknown	^42^	DMG400000711
*AtDMR6*	P. *infestans* / potato *B. cinerea* / tomato Downy mildew / *A. thaliana*	Encodes a salicylic acid 5-hydroxylase that fine-tunes salicylic acid homeostasis	Chilling stress tolerance (tobacco, tomato)	^12,43–45^	DMG400000582 (here denoted *StDMR6-1*) and DMG401026923 (here denoted *StDMR6-2*)

## Materials and methods

### Materials

Tetraploid *Solanum tuberosum* Désirée and King Edward (susceptible to late blight infection) were maintained in vitro by sub-culturing the apical portion of 3–4 week-old stems on Murashige and Skoog (MS) basal nutrient including vitamins (Duchefa, M0222.0050) with 10 g/L sucrose and 7.5 g/L Phyto agar (MS10)^24^. Genetically modified lines containing three resistance genes, *3R, Rpi-blb2, Rpi-blb1, and Rpi-vnt.1*^7,24^, in Désirée and King Edward were used as resistant controls. The *P. infestans* strain 88,069 (A1 mating type, race 1.3.4.7) was propagated as previously described^25^.

### Vector constructs

Candidate genes were selected (Table 1) and the coding sequence analysed for possible CRISPR targets and their number of off-targets using Cas-designer (http://www.rgenome.net/cas-designer);^26^ and CRISPOR (https://crispor.org);^27^. For each candidate, two PCR primer pairs were designed to amplify a region containing putative targets with the fewest potential off-targets and used in PCR amplification of genomic DNA and cDNA (see Supplementary Table). PCR products were run on 1% agarose gels, gel-purified, and each band was sequenced using two primers. For each candidate, the two targets that were conserved in all sequences, and that had the lowest number of potential off-targets were selected (see supplementary Fig. 1). The targets were assembled into the Csy4 multi-gRNA vector pDIRECT_22C, using protocol 3A^22^ to form the plasmid pDIRECT_22C_S-gene.

**Figure 1:**
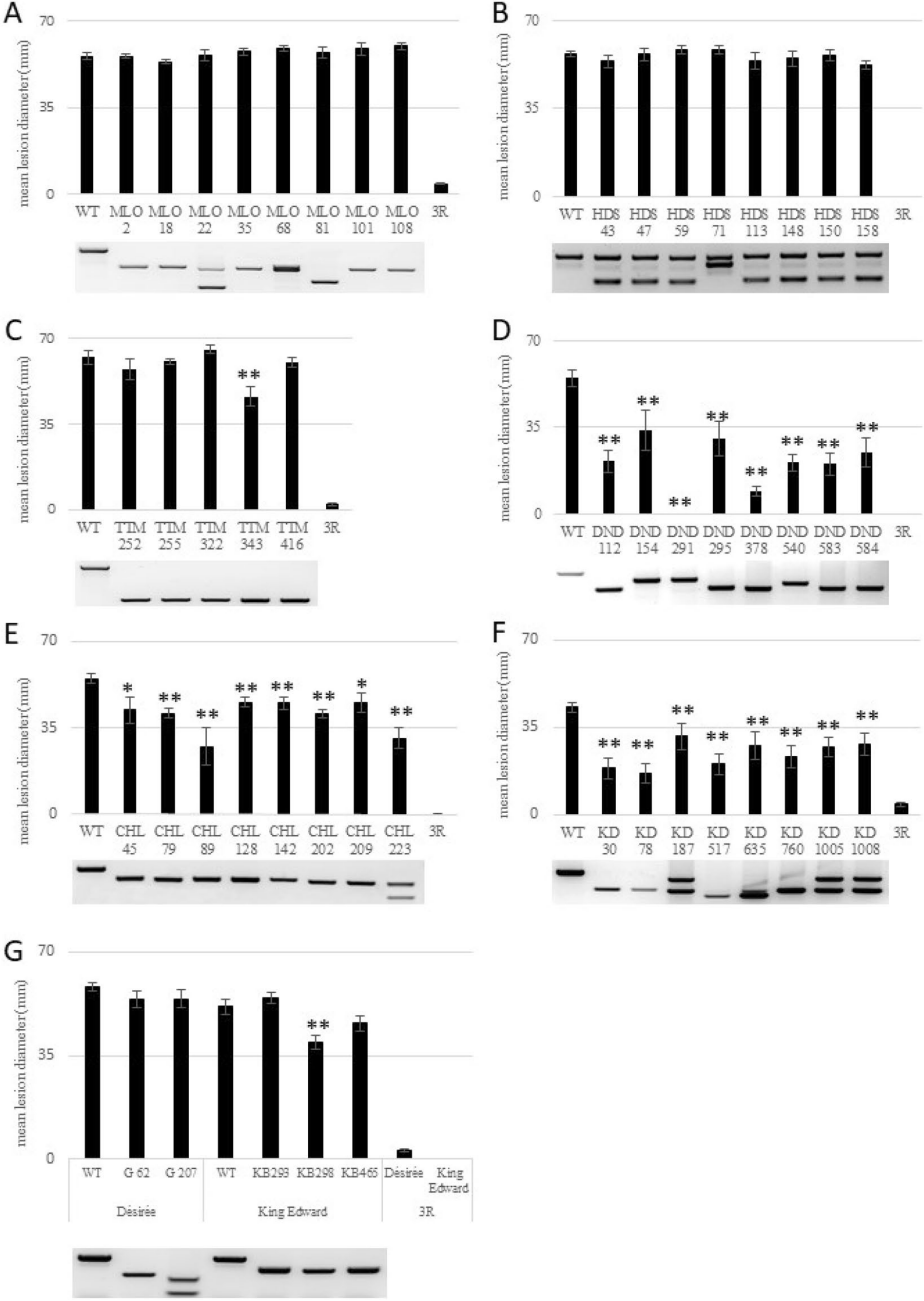
Mean lesion diameter and PCR analysis of potential S-gene mutant lines in potatoes. Lesions caused by *Phytophthora infestans* strain 88069 were scored after 7 d and PCRs were performed with specific primers (Supplementary Table S1) and run in 2% agarose. (**A**) StMLO1. (**B**) StHDS. (**C**) StTTM2. (**D**) StDND1. (**E**) StCHL1. (**F**) StDMR6-1. (**G**) StDMR6-2. Error bars shown represent SEM (standard error of the mean) and asterisks denote values significantly different from that of the wild type (*: *p* < 0.05, **: *p* < 0.01, t-test, n = 9).

### Potato transformation protocol

The protocol for the *Agrobacterium* transformation of *S. tuberosum* Désirée and King Edward was modified from the original protocol^24,28^. A 10 mL overnight liquid culture of *Agrobacterium tumefaciens* C58 carrying the plasmid of interest was centrifuged at 5000 rpm in a 15 ml tube for 10 min, the supernatant was discarded, and the pellet was re-suspended in 10 mL dH_2_O containing 50 µl of acetosyringone (76 mM). For transformation, 1 mL of the *Agrobacterium* suspension (OD 1.9–2.0) was pipetted onto dissected leaf explants that were placed on the co-cultivation media. Leaf explants were incubated under reduced light (50% intensity) for 48 h before they were transferred to selective media (400 mg/L cefotaxime + 100 mg/L kanamycin, and 2 mg/L for Désirée and 5 mg/L for King Edward of zeatin ribose) for regeneration^24^. Leaf explants were sub-cultured onto fresh media every 7–10 d to maintain selection pressure. Shoots that emerged after 4–5 weeks were dissected and rooted on MS media containing no plant growth regulators but with continued selection (100 mg/L kanamycin). Only shoots that initiated roots in the selective media were screened at the molecular level.

### PCR screening and sequencing

Genomic DNA was extracted from young leaves of regenerated potato shoots^29^ and used as a template in the PCR analysis. The PCR reaction mixture contained 1 × Buffer, 1 µL genomic DNA, 0.2 mM dNTPs, 0.5 μM of each primer, and 0.2 U Taq DNA polymerase (Thermo Fisher Scientific, Waltham, USA) in a final volume of 25 µL. The PCR amplification program was as follows: one cycle of 5 min at 95 °C followed by 35 cycles of 20 s at 94 °C, 20 s at 58 to 64 °C (see table S1), and 30 s at 72 °C, with a final extension at 72 °C for 5 min. The samples were analysed on 2% agarose gels (except the *CHL* gene, 3% agarose gels were used) and tetra-allelic deletion mutant lines were selected (except the HDS gene, see results). Each PCR band was isolated from agarose gels and purified using a GeneJET Gel Extraction Kit (Thermo Fisher Scientific, Waltham, USA). Purified samples were sequenced at Eurofins Genomics (Germany), see supplementary figure S2.

### In-vitro propagation and in-vitro long-term storage

Selected mutant lines were propagated by cutting node segments and culturing them in 90 × 25 mm Petri dishes containing 25 mL MS10 medium. The plates were sealed with micropore medical sealing tape and grown in a tissue culture room (20 °C, 16 h photoperiod, 40–60 μmol/m^2^/s). After 14 d, three rooted plants (for each mutant line) were transferred onto the soil for further analysis. To maintain each line in vitro, 1 to 2 shoots were transferred into a Petri dish containing MS10 medium, sealed with Parafilm, cultured for 4 weeks in a tissue culture room; thereafter, the in-vitro line was maintained at 9 °C, 8 h photoperiod, 10 μmol/m^2^/s for 6 months^30^.

### Growth phenotype study and generation of leaf material for pathogenic resistance assay

In-vitro plants of the wild type, 3R, and tetra-allelic deletion mutant lines were grown in 2 L plastic pots containing potting soil (Emmaljunga Torvmull AB, S 28,022 Vittsjö, Sweden). All plants were grown for 5 to 6 weeks in climatized rooms (20 °C, 16 h photoperiod, 160 μmol/m^2^/s, 65% relative humidity [RH]) with watering every second day^31^.

### Detached-leaf assay

For each experiment, nine fully developed leaves from 5-week-old plants from each line were used for detached-leaf assays (DLAs). The inoculum of *P. infestans* was prepared by harvesting sporangia from 12 to 14 d-old plates of *P. infestans* in clean tap water^32^. The inoculum was adjusted to 20,000 sporangia/mL and 25 µL of the spore solution was pipetted onto the abaxial side of the leaflet. The infected leaves were maintained in a humid environment (RH ~ 100%) under controlled conditions^33^. Results were recorded by measuring the infection size of each leaflet at 7 d post-inoculation (dpi). The difference between the means was tested using a t-test with the significance level of *p* < 0.05 or 0.01. We also calculated the percentage of successful infection.

## Result and discussion

### Selection of putative S-genes in Potato against Phytophthora infestans

S-genes involved in susceptibility to different types of pathogens have been found in many different plant species^17,34^. Here, S-gene candidates were selected based on the following criteria: pathogen resistance phenotype, being either a single gene or belonging to a small confined gene family in potatoes, each S-gene concerning other candidates should have a different function, and if possible, function in different pathways (see Table 1).

*MLO* (Mildew resistance locus) encodes a plasma membrane-localized seven transmembrane domain protein associated with vesical transport and callose deposition^8,9,35^. The MLO protein contains a domain that is predicted to bind with calmodulin and is required for full susceptibility to powdery mildew infection^9^. In this study, we included *MLO* because it is a typical S-gene, which has been successfully applied in many plants, such as roses, peas, melons, and apples^9^. Furthermore, *mlo* mutants also showed resistance to two oomycetes: the hemibiotrophic *Phytophthora palmivora*^10^ and the biotrophic *Hyaloperonospora arabidopsidis*^36^. Because *P. infestans* also is a oomycete with a hemibiotrophic lifestyle, we decided to include this gene in the screening. Appiano et al. (2015) identified the corresponding *MLO* gene in potatoes and named it *StMLO1*^37^.

In *Arabidopsis*, *HDS* encodes a chloroplast localized hydroxy-2-methyl-2-(E)-butenyl 4-diphosphate synthase, one of the last steps in the methylerythritol 4-phosphate (MEP) pathway from which chlorophyll, carotenoids, gibberellins, and other isoprenoids are derived^38^. HDS is a negative regulator of salicylic acid (SA) by reducing the amount of its substrate, methylerythritol cyclodiphosphate (MEcPP)^46^. *Arabidopsis HDS* mutant plants show enhanced resistance to biotrophic, but not to necrotrophic, pathogens^47^. In potatoes, we only encountered one *HDS* gene homologue.

The triphosphate tunnel metalloenzymes (TTMs) hydrolyse organophosphate substrates^39^. *Arabidopsis* encodes three TTM proteins, where TTM2 is involved in pathogen resistance via an enhanced hypersensitive response and elevated SA levels^48^. *Atttm2* mutant lines showed enhanced resistance to the biotrophic pathogen *Hyaloperonospora arabidopsidis*. The closest potato homologues to the *AtTTM2* gene are DMG400025117 and DMG400001931. DMG400025117 appeared to be induced by the SA homologue BTH, whereas DMG400001931 was not (http://bar.utoronto.ca/efp_potato/cgi-bin/efpWeb.cgi); therefore, we chose to analyse DMG400025117 since late blight resistance is influenced by SA. Furthermore, as *TTM2* has only been studied in *Arabidopsis*, its relevance in acquiring resistance in crop plants is unknown.

Sun et al. (2016, 2017) analysed potato plants, where *StDND1* had been knocked-down using RNAi and found that the plants were more resistant toward *P. infestans*. *StDND1*-silenced plants displayed auto-necrotic spots only in the leaves of older plants and a few well-silenced *StDND1*-transformants showed dwarfing^12^, a phenotype that might result from inadequate specificity of the RNAi approach or the efficiency of silencing may fluctuate during development. The *DND1* gene encodes a cyclic nucleotide-gated ion channel, which has been implicated in Ca^2+^ signalling related to various physiological processes (pathogen defence, development, and thermotolerance)^49^.

*StCHL1* is a putative S-gene in potatoes. Originally, *StCHL1* was found through microarray analysis of brassinosteroid responsive marker genes in potatoes. Gene overexpression and virus-induced gene silencing experiments showed this gene to be important for *P. infestans* colonization of *Nicotiana benthamiana*^42^. No experiments in potato has been carried out. CHL1 is a transcription factor, which regulates brassinosteroid hormone signalling and immune response^50^; in potatoes, we located only one such gene.

DMR6 proteins belong to the 2-oxoglutarate (2OG)-Fe (II) oxygenase family. In *Arabidopsis*, *AtDMR6* encodes an SA 5-hydroxylase that regulates SA homeostasis by converting SA to 2,5-DHBA^45^. This gene is a negative regulator of the active SA pool; thus, it is important for the SA-dependent plant immune system. Knockout of *SlDMR6-1* in tomatoes enhanced the resistance to *Phytophthora capsici* and *Pseudomonas syringae*^43^. Two DMR6 homologues were identified in potatoes. Knockdown of *StDMR6* in potatoes by RNAi showed an unclear resistance phenotype, with only six out of 12 transformed plants showing lower transcript levels of DMR6 and four plants showed a resistance phenotype, whereas eight plants showed susceptibility to *Phytophthora infestans*^12^. Therefore, both potato *DMR6* homologues were investigated separately by knockout experiments with genome editing.

### Efficiency of double guide mediated tetra-allelic mutation varied between genes

By applying two guide RNAs, targeted deletions in the gene of interest may be generated^22,23^. In a study by Čermák el al. 2017, deletions between the two cleavage sites were far more prevalent than individual indels resulting from cleavage of a single site. Therefore, we used the pDIRECT_22C vector^22^ encoding two guide RNAs for knocking out S-genes in potatoes. For our screen of edited potato plants, we chose to use PCR with gene-specific primers, spanning both gRNA targets, followed by gel electrophoresis analysis, as a simple, inexpensive, and rapid method for detecting deletions in the target gene. The screening results are shown in Fig. 1 for the lines that were subsequently screened for late blight resistance and growth phenotypes. Sequence data of the target regions is shown in supplementary figure S2.

The number of plants with a deletion in all four alleles was related to locus and target sequence (Table 2). Analysis of shoots showed variation in the prevalence of tetra-allelic deletion mutants ranging from 0 to 18%. This number can be regarded as the minimum number because we did not detect single nucleotide mutations with this PCR method, but because it was easy to generate many lines in potatoes we believe this was the most efficient method. Analysing in silico target efficiency with several different online tools did not reveal a specific tool that could predict the mutation rate better than others (Table 2).

**Table 2:** Summary of screening of deletion mutants in this study.

Gene name	*StMLO1*	*StHDS*	*StTTM2*	*StDND1*	*StCHL1*	*StDMR6-1*	*StDMR6-2*	
Potato variety background	Désirée	Désirée	Désirée	King Edward	Désirée	King Edward	Désirée	King Edward
No. of Plants show 4 allele deleted	20 (13%)	No (0%)	5 (1.1%)	14 (2.4%)	39 (18%)	9 (0.7%)	2 (0.9%)	4 (1.2%)
No. of Plants show wild-type band and deleted band	32 (20%)	23 (14%)	74 (15%)	9 (1.5%)	127 (58%)	138 (11%)	50 (23%)	43 (13%)
No. of Plants show only wild-type band	108	145	401	572	55	1124	166	276
Total lines used for screening	160	169	480	595	221	1271	218	323
Guides	TAGCCATAAGGCTAACCATG and TGGCAACAGCTCTTAGAAGC	TATTATGGGGACTCGCCTA and ACGCCTGAACCATAACTAC	CTAGCTCTCGCATAGGATAC and TACGGGATATACAGCGTGCC	AAAGGGACGGCGTAAGCACC and AGCAGCCCAGGTTCTCCAAT	TTGTTCTCCATACAGGGGTC and CCAGTTGGAGTTGGACACGG	GAGAAAATGCTAGGGGTAGC and AGACTTCATTGTCATCCTC	CAGGGGCATATTTGTCCAA and GGTGTATCAAAGAAGGTTA	CAGGGGCATATTTGTCCAA and GGTGTATCAAAGAAGGTTA
CRISPOR Moreno-Mateos score	59 and 41	66 and 25	35 and 67	50 and 50	46 and 84	35 and 43	69 and 60	69 and 60
CRISPOR Doench score	69 and 55	51 and 50	50 and 67	38 and 56	42 and 69	48 and 45	41 and 59	41 and 59
CISTROME	0,21 and 0.27	0.02 and -0.09	-0.1 and -0,32	0.33 and -0,40	-0.31 and 0.83	-0.57 and -0.36	0.69 and 0.03	0.69 and 0.03
Cas-Designer Score (RGEN)	67 and 73	70 and 60	56 and 57	58 and 53	53 and 46	59 and 54	65 and 65	65 and 65
CRISPRater score (CCTop)	0.59 and 0.52	0.75 and 0.58	0.74 and 0.61	0.73 and 0.64	0.64 and 0.68	0.49 and 0.53	0.79 and 0.6	0.79 and 0.6

In *Arabidopsis*, homozygous mutation of HDS caused an albino phenotype and seedling lethality^38^. In the present study, in agreement with this observation, some calli turned white and did not develop into seedlings. Furthermore, none of the *StHDS* genome-edited seedlings were confirmed to be deleted in all four alleles. Therefore we concluded that, as in *Arabidopsis*, a full tetra-allelic HDS deletion is lethal, although transformed cells with a mutation in one, two, or three alleles were able to develop and form shoots (Table 2).

For all other genes, full allelic knockouts were not linked with lethality. Two genes showed a high number of tetra-allelic deletion mutants, namely 13% of *StMLO1* and 18% of *StCHL1* shoots had a deletion in all four alleles. The other four genes showed a prevalence of between 0.7% and 2.4% tetra-allelic deletion mutants. As mentioned above, because the applied PCR screening did not detect point mutations or very short deletions/insertions, the number of mutants detected in the present study may be lower than that of other screening methods, such as CAPS (Cleaved-Amplified-Polymorphic-Sequence) or IDAA^19^. However, a combination of constructs expressing two gRNAs with PCR screening of shoots is a low-cost, simple, and fast method enabling large scale screening at the shoot level (Fig. 1, supplementary Fig. 3).

### StDND1, StCHL1, and StDMR6-1 tetra-allelic deletion mutants showed enhanced late blight resistance

To analyse late blight resistance in tetra-allelic mutant lines, DLAs were performed. Infection lesion diameter was determined 7 days after *P. infestans* inoculation (Fig. 1) and the percentage of infected leaves was analysed (Table 3).

**Table 3:** Percent of successfully infected leaflets in detached-leaf assay.

Gene\line	WT	Mut-1	Mut-2	Mut-3	Mut-4	Mut-5	Mut-6	Mut-7	Mut-8	3R
StMLO1	100	100	100	100	100	100	100	100	100	0
StHDS	100	100	100	100	100	100	100	100	100	0
StTTM2	100	100	100	100	100	100	NA	NA	NA	0
StDND1	100	100	67	0	89	78	89	100	67	0
StCHL1	100	87	100	67	100	100	100	100	78	0
StDMR6-1	100	44	33	33	11	33	22	22	44	0
StDMR6-2 (Desiree)	100	100	100	NA	NA	NA	NA	NA	NA	0
StDMR6-2 (King Edward)	93	87	96	78	NA	NA	NA	NA	NA	0

Mut-1 to Mut-8 are mutant lines and correspond to the lines in Fig. 1 (from left to right). Leaflets from 5-week-old plants were inoculated with 25 μL 20,000 sporangia/mL. Results were scored 7 dpi and a total of nine leaflets per line were used.

**Figure 2:**
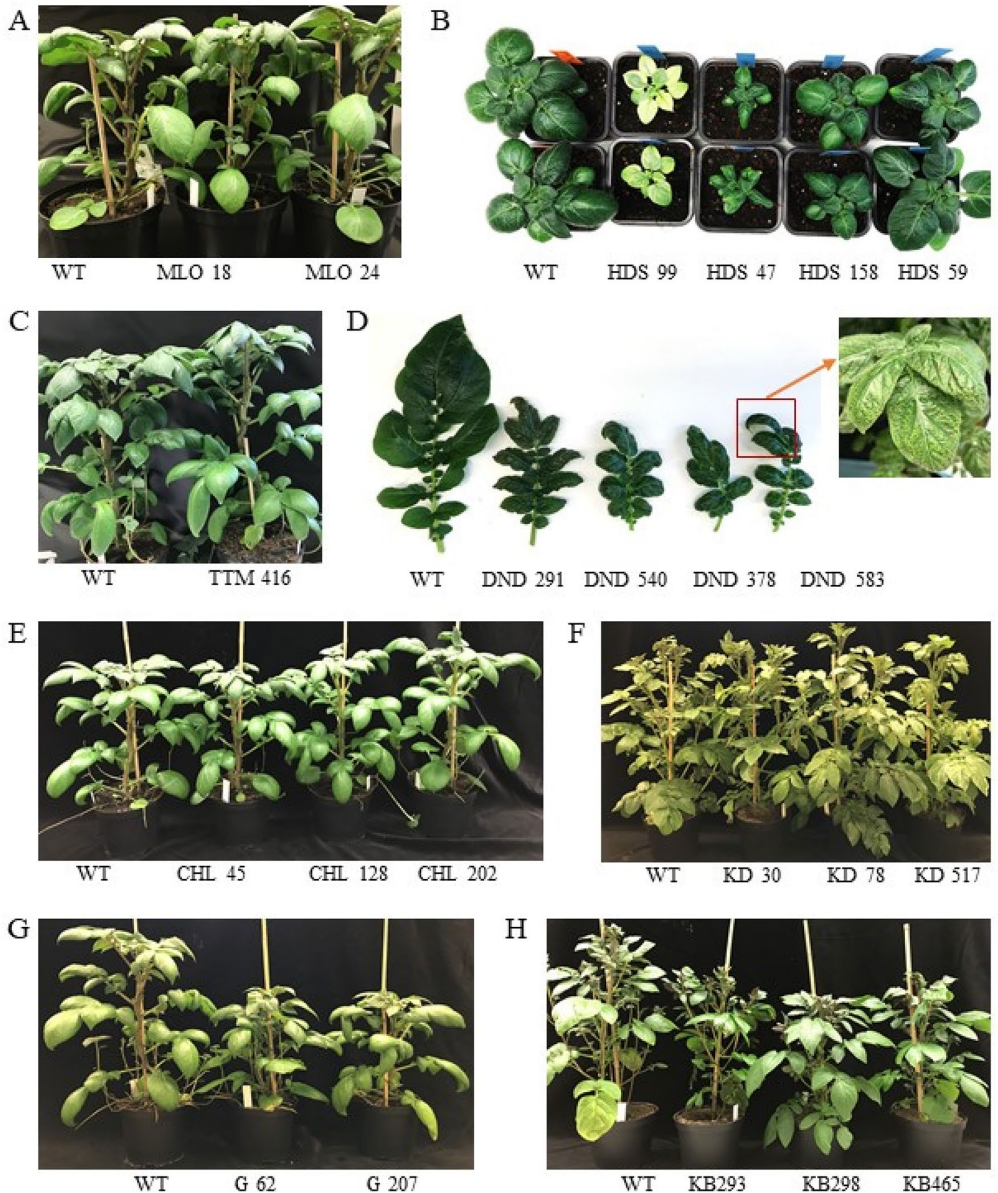
Phenotypes of mutant lines. (**A**) *mlo* at 5-weeks old stage. (**B**) Wild type Désirée and hds mutant lines at 2-weeks-old stage. (**C**) Wild type Désirée and one *Stttm2* mutant line. (**D**) Leaf phenotype of wild type and some *Stdnd1* mutant lines. (**E**) Désirée and* Stchl1* mutant lines at 5 weeks old. (**F)** Wild type King Edward and *Stdmr6-1* mutant lines at 5 weeks old. (**G**) Five weeks old wild type Désirée and *Stdmr6-2* mutant lines. (**H**) Wild type King Edward and *Stdmr6-2 *mutant lines 5 weeks old.

Knockout of *S*t*MLO1* in potatoes did not increase late blight resistance as evident by the sizes of the lesion or percentage of infected leaves. Nor there any growth phenotype was detected (Fig. 2A). The effect on *P. infestans* infection in *mlo* potatoes was tested in the present study for the first time. All eight *Stmlo1* mutant lines were as susceptible to late blight disease as the wild type Désirée (Fig. 1A, Table 3). This was somewhat unexpected because the mutation of orthologous *MLO* genes is effective in many plant and pathogen species^36,37^, including the hemibiotrophic *P. palmivora*. Silencing of *Capsicum annum CaMLO2* conferred enhanced resistance against virulent *Xanthomonas campestris*, whereas overexpression of *CaMLO2* in *Arabidopsis* conferred enhanced susceptibility to both *Pseudomonas syringae* and *Hyaloperonospora arabidopsidis*^36^. Recently, a wheat *mlo* mutant was shown to be susceptible to the hemibiotrophic fungal pathogen *Magnaporthe oryzae,* whereas it was still resistant to the obligate biotrophic fungus *Blumeria graminis*^11^. Thus, the usefulness of MLO is dependent on the host as well as the pathogen.

After PCR screening of 169 putative HDS shoots, we did not obtain any tetra-allelic mutant lines (Table 2). After 2 weeks in soil, some heterozygous mutants showed an albino phenotype (Fig. 2B) and did not grow further, whereas shoots with green leaves grew into adult plants. In *A. thaliana,* the *Athds* was mutagenized with ethyl methanesulfonate (EMS) and influenced chloroplast development and increased resistance to *Pseudomonas syringe*^47^*.* Our potato *Sthds* mutants showed weakened growth (Fig. 2B) and *P. infestans* screening of eight mutant lines did not show increased resistance to late blight disease (Fig. 1, Table 3).

For *StTTM2* (DMG400025117), we analysed five tetra-allelic deletion mutant lines. No mutant line showed any altered phenotype (growth, morphology, or pathogen resistance) when compared with wild-type plants (Figs. 1C, 2C). Analysing *TTM2* sequences in *Solanum tuberosum*, two different *StTTM2* genes were identified (DMG400025117 and DMG400001931). The study of Ung et al. (2017) suggested that *AtTTM1* and *AtTTM2* could functionally complement each other; thus, it is plausible that these genes could be functionally complementary to each other and that a double mutant would show resistance to *P. infestans* in potatoes.

Sun et al. (2016 and 2017) used RNAi to knockdown potato *StDND1* and found that these plants were more resistant to *P. infestans*. However, the plants were smaller and showed early senescence and necrotic spots on leaves of older plants. In line with their results, our data showed that the size of infection lesions was strongly reduced in all *Stdnd1* mutant lines, whereas the percentage of successful infections was reduced in some of the tetra-allelic lines (Fig. 1C and Table 3). Two mutant lines with wild type and mutant PCR-bands (DND 44, DND 82) showed auto-necrotic spots and late blight resistance in older, but not young leaves (Figure S4B and S4C).

The tetra-allelic *Stdnd1* mutated potato not only exhibited a late blight resistance phenotype (Fig. 1D) as observed from the results of the earlier RNAi study but also showed pleiotropic phenotypes, such as line DND 583 (Fig. 2D). The tetra-allelic *Stdnd1* mutant lines, except for the strong resistance phenotype, also showed reduced growth, long and thin stems, as well as necrosis of all leaves (Figure S4A). These latter pleiotropic phenotypes were not found in *StDND1* RNAi lines^12^ maybe because of incomplete silencing. The phenotypes of some of our *Stdnd1* mutants (DND 44 and DND 82) and *StDND1* RNAi lines were very similar (Figure S4 and Fig. 3C of Sun et al. 2016). In summary, our results indicated that *StDND1*, due to the pleiotropic phenotypes observed in the *Stdnd1* edited lines, was not a good candidate for application in agriculture.

**Figure 3:**
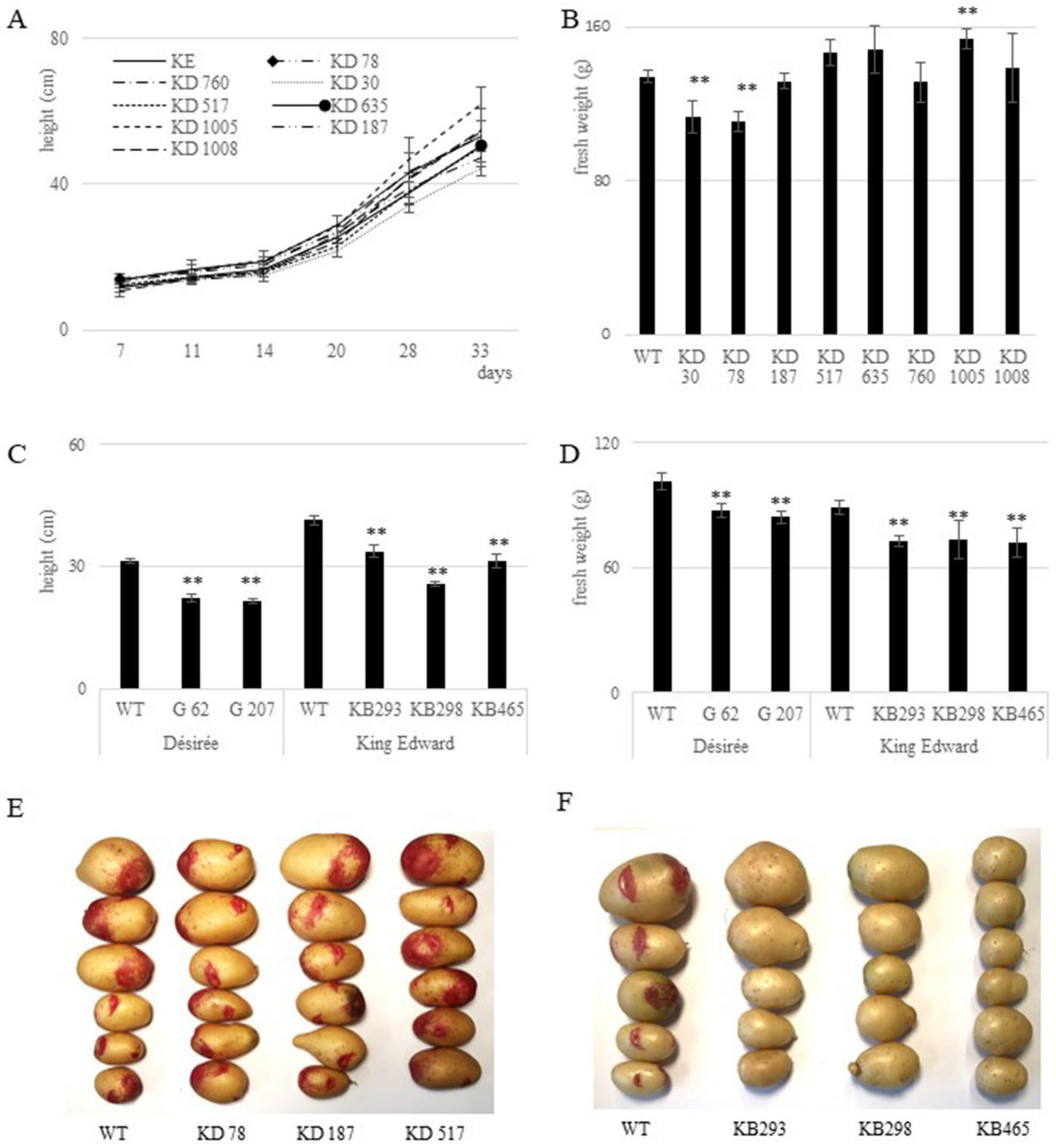
Growth phenotypes of *Stdmr6-1* and *Stdmr6-2* mutant lines. (**A)** Growth curve of wild type and *Stdmr-1* mutant lines. (**B**) Fresh weight of 5-week-old wild type and *Stdmr6-1* mutant lines. (**C**) Plant height of wild type and *Stdmr6-2* mutant lines. (**D**) Fresh weight of 5-weeks-old wild type and* Stdmr6-2* mutant lines. (**E**) Tuber morphology of King Edward wild type and its *Stdmr6-1* mutant lines. (**F**) Tuber morphology of King Edward wild type and *Stdmr6-2* mutant lines. Error bars show standard variation and asterisks denote values significantly different from that of the wild type, student t-test (**: *p* < 0.01, n = 4 for King Edward and n = 6 for Désirée).

*Stchl1* mutations did not affect morphology or growth phenotype (Fig. 2E). Tetra-allelic mutant plants showed a significant late blight resistance phenotype with reduced lesion sizes (Fig. 1E), but no difference in the percentage of infected leaves (Table 3). This could indicate that the importance of this protein is at the disease developmental stage and not in the initial phase. With a function as a *Phytophthora* effector target and transcription factor, and being involved in brassinosteroid hormone signalling and immune response to *P. infestans*^50^, *StCHL1* has clear potential as an useful S-gene; possibly when combined with other S- or R-factors to improve pathogen resistance.

CRISPR/Cas9 was applied to knockdown both *StDMR6-1* and *StDMR6-2,* respectively. Tetra-allelic CRISPR/Cas9 knockdown of *StDMR6-1* showed a significant increase in resistance against *P. infestans* both as measured by infected lesion size and the percentage of infected leaves (Fig. 1F, Table 3). This is in contrast to that of *Stdnd1* and *Stchl1* knockout plants, which only showed reduced infection lesion sizes (Fig. 1 and Table 3), but no reduction in the percentage of infected leaves. In tomatoes, the CRISPR-Cas9 mediated mutation of the *StDMR6-1* ortholog *SlDMR6-1* showed increased resistance to *P. capsici* and *P. syringae* pv. tomato^43^, indicating broad-spectrum disease resistance function of DMR6-1. In potatoes, knockdown of *StDMR6* by RNAi increased late blight resistance without any documented effect on growth phenotype^12^. However, only 33% of the RNAi lines showed an increased resistance phenotype^12^. Tomatoes and potatoes each contain two DMR6 genes (^43^, Table 1). *StDMR6*-2 and *StDMR6-1* transcripts are approximately 80% identical at the nucleotide level. Because these genes are remarkably similar, RNAi may downregulate both, and therefore knock out of either gene by CRISPR-Cas9 is important for the elucidation of individual gene function.

Genome editing of *StDMR6-2* showed that this gene was not involved in susceptibility to *P. infestans* (Fig. 1G and Table 3). Five tetra-allelic mutants in two potato backgrounds (Désirée and King Edward) showed the same infection lesion size and percentage of infected leaves as that of the wild type. De Toledo Thomazella et al. (2016) did not study tomato SlDMR6-2 further because of the low expression during pathogen infection.

In conclusion, when comparing the DLA results of mutant lines with both wild type (Désirée and King Edward) and an R-gene containing a transgenic line (3R), we identified three genes (*StDND1, StCHL1,* and *StDMR6-1*) that when mutated, increased late blight resistance, whereas mutations in *StMLO1*, *StHDS*, *StTTM2,* and *StDMR6-2* did not affect late blight resistance in potatoes.

### DMR6-1 mutants had no obvious growth-related phenotypes

*StDMR6-1* is a promising S-gene because tetra-allelic mutants not only showed increased late blight resistance (Fig. 1F and Table 3) but also did not differ in over-all growth phenotype compared with the wild type (Fig. 2F). Measurement of plant height (Fig. 3A), fresh weight (Fig. 3B) and tuber morphology (Fig. 3E) showed no differences between mutants and wild types. Plants mutated in the orthologous gene *SlDMR6-1* in tomatoes, showed disease resistance without any documented effects in growth and development under greenhouse conditions^43^. Therefore, StDMR6-1 may be used in potato breeding to create new potato cultivars with broad-spectrum disease resistance.

### StDMR6-2 affect growth phenotypes in potato

*St*DMR6-1 and its ortholog SlDMR6-1 are important in pathogen susceptibility (Fig. 1)^43^ without any obvious growth phenotype (Fig. 3). We investigate the effect of the genome editing of StDMR6-2 on potato phenotype (Figs. 2G,H and 3). Our results did not show any changes in late blight resistance. Analysis of growth phenotype showed that tetra-allelic mutants of *St*DMR6-2 had significantly lower plant height (Fig. 3C) and fresh weight (Fig. 3D) in both cultivar backgrounds. The plants had the same number of leaves as did the wild type, but their internodes were shorter (Fig. 2G). Furthermore, the tuber eyes of *St*DMR6-2 mutants did not have the reddish colour (anthocyanin) that is typical of King Edward (Fig. 3F). Moreover, analysis of amino acid domain of StDMR6-2 showed that StDMR6-2 belonged to the 2-oxoglutarate (2OG)-Fe (II) oxygenase family proteins, which are well known for the regulation of secondary metabolism and plant hormones^51^. Therefore, we hypothesize that *St*DMR6-2 may function in plant secondary metabolism (anthocyanidin) and may not be involved in late blight resistance. *St*DMR6-1 and *St*DMR6-2 share 80% homology at the amino acid level. The nearest solved structure is anthocyanidin synthase from arabidopsis thaliana complexed with naringenin (https://www.rcsb.org/structure/2brt), which when superimposed with *St*DMR6-1 or *St*DMR6-2 yields reliability scores^52^; http://www.cbs.dtu.dk/services/CPHmodels/) too low to allow for structure prediction/comparison, which could shed light on potential substrate/functionality differences between S*t*DMR6-1 and *St*DMR6-2.

## Conclusion

Using CRISPR-Cas9 mediated loss of gene function of seven putative S-genes, we showed that three putative S-genes (*StDND1, StCHL1,* and *StDMR6-1*) were involved in late blight susceptibility. Among these three, *StDMR6-1* and *StCHL1* emerged as promising S-gene targets for the breeding of new disease resistance cultivars because they did not show any growth related phenotype. We also concluded that the pDIRECT_22C vector and the applied deletion screening system expressing two gRNAs for fast PCR mediated screening of full or partial allele knockout was highly efficient and applicable in potatoes. We have produced gene-edited material in popular cultivars that are ready for further tests in field trials.

## Supplementary Information

Supplementary Information
