# Supplementary material for: Mutations introduced in susceptibility genes through CRISPR/Cas9 genome editing confer increased late blight resistance in potatoes
Source: Sci Rep. 2021 Feb 24;11:4487. doi: 10.1038/s41598-021-83972-w (PMC7904907; doi:10.1038/s41598-021-83972-w)
Supplement: Supplementary file 1 — Supplementary Information [file 41598_2021_83972_MOESM1_ESM.docx]

**MUTATION INTRODUCED IN SUSCEPTIBILITY GENES THROUGH CRISPR/CAS9 GENOME EDITING CONFER INCREASED LATE BLIGHT RESISTANCE IN POTATOES**

Nam Phuong Kieu, Marit Lenman, Eu Sheng Wang, Bent L Petersen, Erik Andreasson

**Supplementary material**

*Figure S1. Schematic representation of genes targeted for editing using CRISPR/Cas9. Black boxes represent exons. Target sites of single-guide RNAs are labelled A and B.*

*
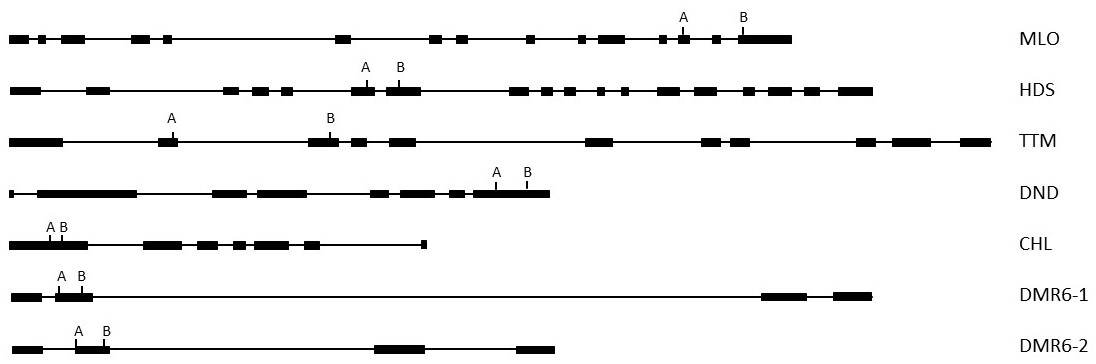
*

*Figure S2. Sequences of mutated lines, corresponding to figure 1. WT is the wild type sequence. a and b refer to sequences from different PCR bands from the same line. Guide-target sequences are underlined. -------- equals deleted regions in mutant lines, ---NNN--- (sequences between ---) show inserted sequence and // denotes sequence regions that is not shown in order for clarity.*

**

*Figure S3. Agarose gel pictures of PCR screeing of mutants. PCR products were run in 2 % agarose gels at 100 V for 1 hour (except CHL mutant, 3 % gel, 150 V and 2 hours). Thermo Scientific GeneRuler 1 kb DNA was used as size marker.*


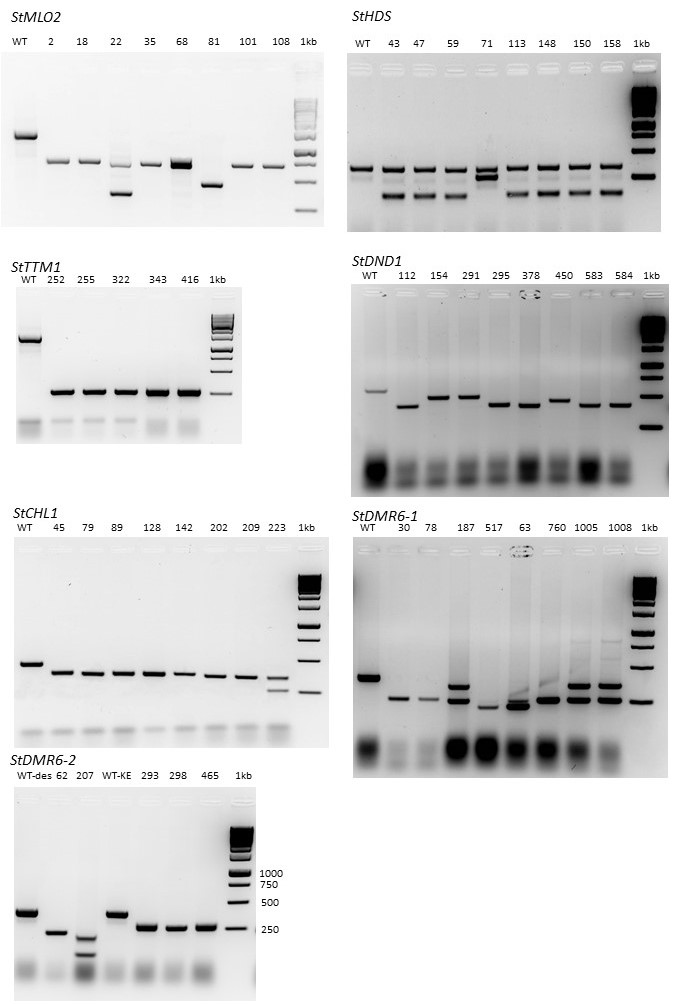


*Figure S4: The phenotype of Stdnd1 mutant lines. A: The tetra-allelic deleted mutant DND 112 showed dwarf phenotype when grown in soil. B:The heterozygous Stdnd1 mutant (DND 44, DND 82) did not have any effect on growth phenotype but had auto-necrotic spots on older leaves. C: The tetra-allelic deleted mutant (DND 291) and the heterozygous Stdnd1 mutant (DND 44) showed different levels of resistance against Phytophtora infestans.*

**
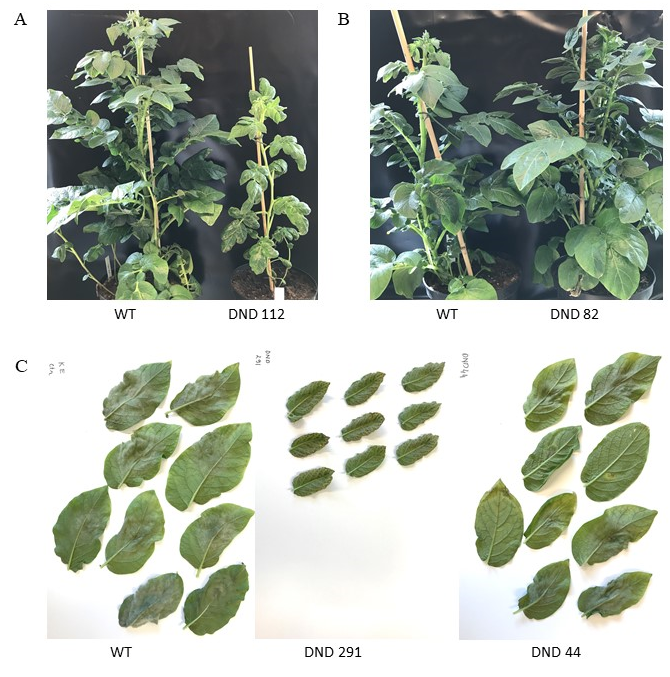
**

*Table S1:* *Gene specific primers used for amplification and sequencing of plant DNA as well as screening of shoots. Primers specific for a potato housekeeping gene (EF1α) and an Agrobacterium gene (virG) were used to confirm absence of Agrobacterium in shoots (Wang et al, 2020). na: not applicable.*

|  |  |  |  |  |  | **PCR analysis of shoots** | | |
| --- | --- | --- | --- | --- | --- | --- | --- | --- |
| Gene | Primer | Sequence | Primer | Sequence | PCR amplification and sequencing | Tm | gDNA WT band, bp | Mutant band, bp |
| StMLO1 | MLO_F3 | AGGATTTCAGAAAGGGGAGACA | MLO_R3 | CGTAATAAGTGAACAGGGGAGG |  | 62 | 1271 | 681 |
| StMLO1 | MLO_F2 | ACTCGTATCTTTGGGTGCCA | MLO_R2 | ATGTCCGAGGAGATGCTTGA | x | 58 |  |  |
| StMLO1 | MLO_F3 | AGGATTTCAGAAAGGGGAGACA | MLO_R1 | TTTGTTTCCAAAAGTGAAATCTGA | x | 58 |  |  |
| StHDS | HDS_F835 | ACCATGGGAGCCTTTCAGA | HDS_R1087 | TCCATCCTCACCTTCACCAG |  | 64 | 341 | 122 |
| StHDS | HDS_F806 | AAGTATGGACGTGCAATGCG | HDS_R1087 | TCCATCCTCACCTTCACCAG | x | 58 |  |  |
| StHDS | HDS_F835 | ACCATGGGAGCCTTTCAGA | StHDS_qPCR_Rev2 | CTGAAGAAGTGTGCCAATAC | x | 58 |  |  |
| StTTM2 | TTM_F3 | CGACTTACAGACTATGATACATTGC | TTM-R2 | ATGTGCAGTCTTGAGATCAGG |  | 60 | 1330 | 280 |
| StTTM2 | TTM_F1 | GCCTAAAGATACTAGTAATGGTGAATC | TTM_R2 | ATGTGCAGTCTTGAGATCAGG | x | 58 |  |  |
| StTTM2 | TTM_F2 | TCTTAAAGGACCAGGTTCGAC | TTM_R1 | TTTGATCGACTTGCACATCC | x | 58 |  |  |
| StDND1 | DND_F1 | ATCGCGTTAAGCCACTTG | DND_R2 | GATTGACAAGAACATTGCAGC | x | 60 | 590 | 410 |
| StDND1 | DND_F2 | AGTGCACAGGGTTGTGTTC | DND_R3 | CGAAGACGATGATCATTATCC | x | 58 |  |  |
| StDND1 | DND_F2 | AGTGCACAGGGTTGTGTTC | DND_R2 | GATTGACAAGAACATTGCAGC | x | 58 |  |  |
| StCHL1 | CHL-F1 | AATGGAGGAAATGGAAGCAG | CHL_R4 | CTTGAAGTTCTCACTGCCTCTG |  | 60 | 435 | 345 |
| StCHL1 | CHL_F1 | AATGGAGGAAATGGAAGCAG | CHL_R3 | AAGAATCATCCCCCTCTTCG | x | 58 |  |  |
| StCHL1 | CHL_F2 | GCTTGAACGACAACCAGCTA | CHL_R2 | TTATTGGCGCTCCTTTTGTT | x | 58 |  |  |
| StDMR6-1 | DMR61_F1 | ACTTAGGTTGCGGAGACCAA | DMR61_R1 | TTACCTGAAAGACGATGGAT |  | 58 | 409 | 237 |
| StDMR6-1 | DMR61_F2 | CGAAAGTTATTTCCAGCGG | DMR61_R1 | TTACCTGAAAGACGATGGAT | x | 58 |  |  |
| StDMR6-1 | DMR61_F1 | ACTTAGGTTGCGGAGACCAA | DMR61_R2 | CAACATCTGATACGATACACACTGT | x | 58 |  |  |
| StDMR6-2 | DMR62_F1 | AGCAACATTACTATTTGGAGGTTTC | DMR62_R1 | TTACCTGAAAGAAGGAGGATT |  | 54 | 381 | 173 |
| StDMR6-2 | DMR62_F2 | CAAGTGTTCTTTCCGGTGG | DMR62_R1 | TTACCTGAAAGAAGGAGGATT | x | 58 | 648 | 440 |
| StDMR6-2 | DMR62_F1 | AGCAACATTACTATTTGGAGGTTTC | DMR62_R2 | TACGCCGTGACAAAGCAA | x | 58 |  |  |
| Potato EF1α gene | St Ef1α F2 | GAACTGTCCCTGTTGGTCGT | St Ef1α R2 | GGGTCATCCTTGGAGTTTGA |  | 58 | 220 | na |
| Agrobacterium virG gene | VirG+ | CGCACGCGCAAGGCAACC | VirG- | GCCGGGGCGAGACCATAGG |  | 58 | 606 | na |
